# Diabetic Retinopathy Detection from Fundus Images of the Eye Using Hybrid Deep Learning Features

**DOI:** 10.3390/diagnostics12071607

**Published:** 2022-07-01

**Authors:** Muhammad Mohsin Butt, D. N. F. Awang Iskandar, Sherif E. Abdelhamid, Ghazanfar Latif, Runna Alghazo

**Affiliations:** 1 Faculty of Computer Science and Information Technology, University of Malaysia, Kuala Lumpur 50603, Sarawak, Malaysia; engr.mohsin.butt@gmail.com (M.M.B.); dnfaiz@unimas.my (D.N.F.A.I.); 2 Department of Computer and Information Sciences, Virginia Military Institute, Lexington, VA 24450, USA; 3 Computer Science Department, Prince Mohammad Bin Fahd University, Khobar 34754, Saudi Arabia; 4 Department of Computer Sciences and Mathematics, Université du Québec à Chicoutimi, 555 Boulevard de l’Université, Chicoutimi, QC G7H 2B1, Canada; 5 College of Sciences and Human Studies, Prince Mohammad Bin Fahd University, Khobar 34754, Saudi Arabia; rghazo@pmu.edu.sa

**Keywords:** hybrid deep learning features, fundus images, diabetic retinopathy, convolutional neural network features

## Abstract

Diabetic Retinopathy (DR) is a medical condition present in patients suffering from long-term diabetes. If a diagnosis is not carried out at an early stage, it can lead to vision impairment. High blood sugar in diabetic patients is the main source of DR. This affects the blood vessels within the retina. Manual detection of DR is a difficult task since it can affect the retina, causing structural changes such as Microaneurysms (MAs), Exudates (EXs), Hemorrhages (HMs), and extra blood vessel growth. In this work, a hybrid technique for the detection and classification of Diabetic Retinopathy in fundus images of the eye is proposed. Transfer learning (TL) is used on pre-trained Convolutional Neural Network (CNN) models to extract features that are combined to generate a hybrid feature vector. This feature vector is passed on to various classifiers for binary and multiclass classification of fundus images. System performance is measured using various metrics and results are compared with recent approaches for DR detection. The proposed method provides significant performance improvement in DR detection for fundus images. For binary classification, the proposed modified method achieved the highest accuracy of 97.8% and 89.29% for multiclass classification.

## 1. Introduction

Diabetes Mellitus (DM) is a group of medical conditions in which the human body ends up with high blood sugar. There can be various causes of high blood sugar, for example, deficiency in insulin production or lack of cell response towards insulin [1,2]. The World Health Organization (WHO) predicted an increase in DM in the near future [3,4]. DR is one complication that occurs because of diabetes. It mostly remains undetected until the later stages of the disease. Hence, its early detection is necessary to prevent vision loss [5,6]. The increased sugar content affects the vessels inside the retinal tissues. Fundoscopy is a medical imaging technique used to capture the internal structure of the retina [7]. The fundus images captured through this technique reveal different retinal structures of the eye. The grading of DR images by an ophthalmologist is a long process that requires meticulous examination. The different abnormalities caused by DR in the eye include red lesions such as Microaneurysm (MA) and intra-retinal hemorrhages. Besides these, white lesions that appear in the eye because of DR include exudates (EX) and cotton-wool spots. A Microaneurysm (MA) is a tiny aneurysm or swelling on the side of a blood vessel [8]. These small aneurysms can weaken the capillary walls, which can rupture and leak blood from the blood vessel. The leaked blood because of a Microaneurysm causes hemorrhages [9] around the blood vessels inside the retina. The cause of vessel damage in the retina is not only limited to diabetes. An excess of reactive oxygen species during active retinal usage and obstructive sleep apnea syndrome can also cause various retinal disorders [10,11]. The abnormalities caused by DR also surface in other molecular and genetic analyses of the retina. These retinal pathologies cause the alteration of specific pathways such as inflammation and vascular alterations [12,13]. There are many traditional image processing and machine learning (ML) techniques that are proposed in the literature for isolating these lesions [14,15]. Support Vector Machines (SVM) are an important technique that helps in the fast and accurate separation of different classes by transforming the input features into hyperplanes using kernel functions [16,17].

Recently, the image processing field has been aided by Convolutional Neural Networks (CNN) [18,19,20]. An end-to-end system requiring minimal preprocessing results from the integration of the various image features and classifiers in CNN. Multiple layers and their depth can greatly affect the enhancement of Feature extraction. It was found that deep learning networks (DL) maximize the performance. However, increasing the depth of the network can introduce various problems such as vanishing gradients and degradation, resulting in high training errors. Different architectures were proposed in the literature to optimize these networks for image classification.

The motivation of this work is to design a system that can automatically detect and diagnose Diabetic Retinopathy (DR) from the eye fundus images using hybrid deep learning features. The manual diagnosis of this medical image is a time-consuming task that requires specialized personnel who have vast experience in diagnosing this eye condition from medical imaging and other factors. This makes this diagnosis an expensive one as well when specialized medical experts are doing the diagnosis. In addition, and due to human limitations, only a limited number of patients can be processed at a given time. The process is also prone to human errors which is sometimes the case in many medical diagnosis procedures performed by doctors. Thus, the term of getting a second opinion is always stressed for individuals who are diagnosed with serious medical issues. Due to all these shortcomings, if it is at all possible to automate this procedure, it will reduce the cost, reduce diagnostic errors, and speed up the process so that many patients can be processed around the clock. With this procedure, we are not calling for the elimination of the specialized doctors, however, the output of such a system will aid these specialists so that they do not spend the same time processing a patient as they would manually.

This work aims to design an automated system that can automatically detect and diagnose DR from eye fundus images using hybrid deep learning features. The system will be trained on a dataset and will be able to detect and diagnose based on any new image from the test dataset. Provided that the detection and diagnosis accuracy is high, the system will be able to assist doctors in the proper diagnosis of this condition while reducing human errors and reducing costs. In addition, the system will be able to tackle any shortcomings of the manual diagnosis, as mentioned earlier. In this work, we propose a hybrid technique that utilizes the pre-trained CNN models of GoogleNet [21] and ResNet-18 [22] to extract features from the fundus images and perform both binary and multiclass classifications of fundus images. 

The rest of the paper is organized as follows. In Section 2, recent methods that are used for detecting DR using the fundus images of the eye are summarized. In Section 3, the proposed hybrid model for DR classification is presented. Section 4 presents the results obtained by applying the proposed model to input data using different performance metrics. Section 5 compares the results and performance of the system with recent approaches for DR classification. In Section 6, the conclusions are presented as well as future work.

## 2. Literature Review

The literature related to DR detection is mostly divided into traditional and modern machine learning and image processing techniques. In the past, the fundus images were pre-processed, and feature extracted using various image processing methodologies. Various traditional machine learning methods were used afterward for the classification of the resulting feature extracted images. These methods were trained and tested on a smaller dataset and required careful extraction of the handcrafted features that accurately represented the data. With the availability of powerful hardware with high processing power and large image datasets, CNN, which is a branch of machine learning modalities, has become widely popular in the feature extraction and classification of medical images.

The authors of the research article [23] use transfer learning by adding CNN layers on top of ResNet and Inception-based models for multi-class classification of fundus images from the Asia Pacific Tele-Ophthalmology Society (APTOS) [24] blindness detection dataset. The images are pre-processed using resizing, blurring, and bounding box operations while data augmentation is performed to balance the data. The authors report a test accuracy of 82.18% on the APTOS dataset.

A multiclass classification approach for different eye-related diseases is proposed in [25]. The method uses CNN architectures and Transfer Learning for fundus image classification into different categories of ocular diseases. The Ocular Disease Intelligent Recognition dataset provided by Peking University contains class labels for eight categories of ocular diseases that are labeled normal, diabetic, Glaucoma, cataract, hypertension, myopia, AMD, and other diseases. The authors propose two models using Transfer Learning (TL). The first one uses right and left fundus images of the eye to create a parallel architecture whose feature vectors are combined before applying the pooling layer at the end. The second architecture uses a concatenated image of the right and left eye of the input for classification. The results show higher performance on the concatenated image input of the second model using transfer learning on the VGG16 CNN architecture.

A coarse-to-fine CNN architecture is proposed in [26] that first uses a coarse network to perform binary classification of the input data into No DR and DR affected images. The architecture introduces attention gate modules into the CNN architecture that reduces background information and enhances the lesion features. The Fine Network later classifies the remaining four stages of DR, i.e., mild, moderate, severe, and proliferative DR from the DR classified images of the coarse network. The datasets used in the paper are the EyePACS (a platform providing DR images of left and right eyes taken from different types of cameras) and the Indian Diabetic Retinopathy Image Dataset (IDRiD). The IDRiD dataset contains images acquired from Kowa VX-10α digital fundus camera from an Eye Clinic in India). The model achieved a maximum accuracy of 83.1% on the EyePACS dataset and 56.19% on the IDRiD dataset.

The work presented in [27] uses deep CNN for the binary classification of the retinal fundus images. They separate the input images into two classes: no DR and referable DR. The referable DR groups the images from stages 1–4 of the International Clinical Diabetic Retinopathy Severity (ICDR) severity scale [28]. The ICDR severity scale groups the images into five different stages of DR depending on the disease progression. The performance metrics used for comparison of the results are area under the curve, specificity, and sensitivity. Noise is removed from the input images in the preprocessing phase and a CNN with nineteen layers is trained for extracting image features and classification. The CNN used is a modified version of the VGGNet CNN architecture proposed in the image classification challenge.

The authors of [29] propose a deep-learning-based model that uses the DenseNet encoder and convolutional attention module block for DR severity detection. The encoder is used to extract the features from the input fundus images from the APTOS dataset and the attention block is used for refining the features. The authors achieved a binary classification accuracy of 97% and multiclass classification accuracy of 82%. Another study [30] introduces artificial synaptic meta plasticity into the initial learning stages of different CNNs for enhancing the feature extraction by the CNN models. They achieved an average accuracy of 94% on binary classification. The authors of [31] design a source-free transfer learning model for the binary classification of DR images. The model achieved a 91.2% accuracy on the APTOS dataset.

The authors in [32] explore the detection of DR from fundus images based on multi-channel CNNs. They reported an accuracy of 97.08% for binary classification. It should be mentioned here that ML and DL algorithms for the detection and diagnosis of medical conditions are not limited to DR but are being explored for various medical conditions and non-medical applications as well. In [33], the authors use a deep neural network-based feature for the classification of Glioma tumors. In [34], the authors utilize an optimized deep learning approach for lung cancer detection. In [35], the authors combine machine learning with the Internet of Things and cloud computing for the diagnosis and medication of ill individuals in their homes.

To summarize, modern approaches for DR detection rely on two fundamental approaches. If the input data are large, a custom CNN can be trained to detect and identify various stages of DR. This takes a long processing time to train the model to extract features and classify the image. However, for smaller datasets that do not have enough information to fully train the CNN, transfer learning is utilized. Transfer learning can speed up the training process and also provide ample features for problems with smaller datasets. Some authors have also combined various stages of DR into either two or three-class classification problems due to interclass similarities that make the model easier to train. Having a lesser number of classes increases the performance of these systems but at the cost of reduced information about class separation. These are just a small sample of the vast research being performed in this field and in particular in the field of medicine. However, machine learning is a research area that is touching on all fields whether business, education, finance, etc.

## 3. Methodology

In this article, we present a hybrid approach using transfer learning based on GoogleNet [21] and ResNet-18 [22] architectures. In preprocessing, the images are resized and normalized to match the input image requirements of the GoogleNet and ResNet-18 Models, i.e., 224 × 224 × 3. We freeze the layers of both architectures and pass the input fundus images through these models. At the end of each architecture, we extract 1000 features from the fully connected layer and remove the SoftMax layer, which is used for classification within these models. Each of these models applies the convolution, normalization, and pooling layers on the input fundus images. GoogleNet uses the inception modules to reduce the computational resources and capture the spatial and local features, while the ResNet-18 model uses skip connections to avoid degradation and reduce the training error. We merge the feature vectors obtained from GoogleNet and ResNet-18 models to form a hybrid feature vector that contains 2000 features. This feature vector is passed to different classifiers and results are compared with different methods for DR classification. For binary classification, the fundus images are grouped into two categories, i.e., no DR (NDR) which represents stage 0 of the ICDR severity scale, and DR images that combine the images from stages 1–4. For multiclass classification, the images are grouped into three classes, i.e., no DR (NDR) representing the images from stage 0 of the ICDR severity scale, MDR representing the images from stages 1 and 2 (mild and moderate), and PDR, which characterizes the stages 3 and 4 (severe and proliferative) of the ICDR severity scale.

**Figure 1:**
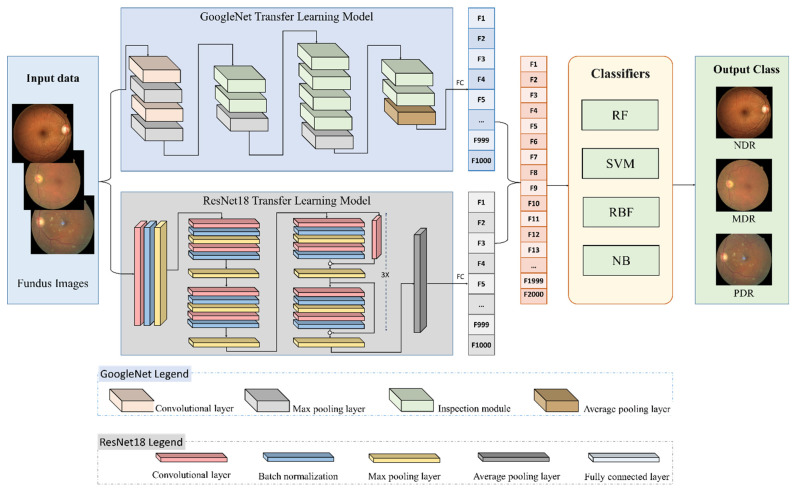
The proposed method for hybrid feature extraction and classification of fundus images.

Figure 1 shows the proposed methodology for the hybrid feature extraction and classification of fundus images. After the preprocessing phase, the images are input to both the GoogleNet and ResNet-18 transfer learning model. A total of 1000 features will be extracted using each model. The combined 2000 features will then be input to well-known classifiers such as the Naïve Bayes (NB), Random Forest (RF), Radial Basis Function (RBF), and Support Vector Machine (SVM). Metrics including Precision, Accuracy, Recall, and F-measure will be used to compare the performance of the classifiers and compare the results achieved in this work with similarly proposed methods in the extant literature.

### 3.1. Experimental Dataset

**Figure 2:**
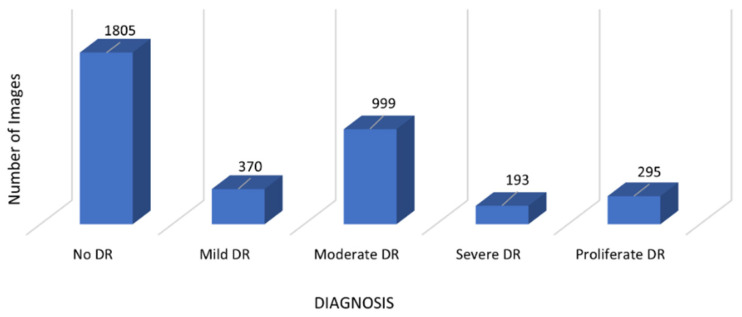
Fundus image distribution of different classes in the APTOS dataset.

In this work, the fundus images used for training the system are from the Asia Pacific Tele-Ophthalmology Society (APTOS) blindness detection dataset. The data are available on the Kaggle website [24]. There are 3662 fundus images present in the dataset that were collected from The Aravind Eye Hospital in India. The labels for the images use the ICDR severity scale for five stages of DR classification, i.e., 0 (No DR), 1 (Mild DR), 2 (Moderate DR), 3 (Severe DR), and 4 (Proliferative DR). The distribution of the data for the various stages of DR is shown in Figure 2.

**Figure 3:**
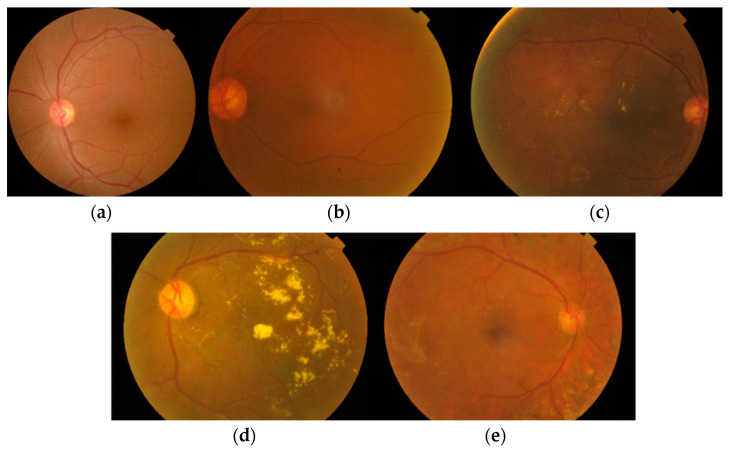
Various stages of DR according to the ICDR severity scale in the APTOS dataset. (**a**). Healthy image with No DR. Stage 0, (**b**). Image with Mild DR. Stage 1, (**c**). Image with Moderate DR. Stage 2, (**d**). Image with Severe DR. Stage 3, (**e**). Image with Proliferative Dr. Stage 4.

A sample of images suffering from various stages of DR according to the ICDR severity scale in the APTOS dataset is shown in Figure 3. The healthy images with no DR contain no Micro Aneurysms (MAs) or Hemorrhages (HEs). Images labeled in stage 1 contain a few lesions. In stage 2, images contain some MAs, exudates (EXs), and at least one hemorrhage (HE). Stage 3 contains MAs from 5 to 15 and HEs less than 5. In the last group with images labeled as stage 4, MAs above 15 and HEs above 5 are present.

**Figure 4:**
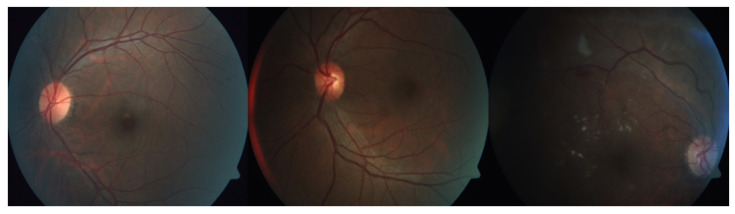
Sample images suffering from focus and exposure issues.

The data suffer from major imbalance and other problems including noise, artifacts, focus, and exposure. These issues are illustrated in Figure 4.

### 3.2. Hybrid Convolutional Neural Network Feature Extraction

Deep learning methodologies are based on the fundamental principles of the Artificial Neural Network (ANN) [36]. The structure of these networks is inspired by the collective working of neurons inside a human. The fundamental element in an ANN is the perceptron whose output we can calculate using Equations (1) and (2).

(1) $$y=f\left(w_{0}+\sum^{\;}X^{T}*W\right)$$

(2) y=f(z)

where

$$z=w_{0}+\sum^{\;}X^{T}*W$$

In the equations above, *X* is the input, *W* is the weight matrix, *w*_0_ is the bias, and *f* is the non-linear function. An ANN consists of multiple neurons whose weights are trained to predict the output from the given input. Another important factor required for the accurate training of the network is the large number of training inputs that can help capture the features of the input. In a multilayer ANN, several layers each containing a collection of perceptrons are used. The perceptron layers are called hidden layers.

A deep neural network consists of multiple hidden layers and needs abundant input data to accurately train the deep network to learn the features of the input. A Convolutional Neural Network (CNN) is a type of deep learning network that is mainly used for processing image data. A fully connected neural network cannot capture the spatial features of an input image. Therefore, in CNN, a convolution operation is performed on the image using various filters (each filter captures a particular image feature, e.g., edge, smoothness, brightness, etc.) to create a feature map. Non-linearity is introduced in the activation layer using a Rectified Linear Unit (ReLU) operator [37]. The hidden layers of an ANN are replaced with convolution layers. The convolution layers capture various low, mid, and high-level features of the input image. Pooling is performed to reduce the dimensions of the input image. In the last stage, the image with the reduced feature set is flattened, and a fully connected layer is used to predict the output classes.

#### 3.2.1. GoogleNet

**Figure 5:**
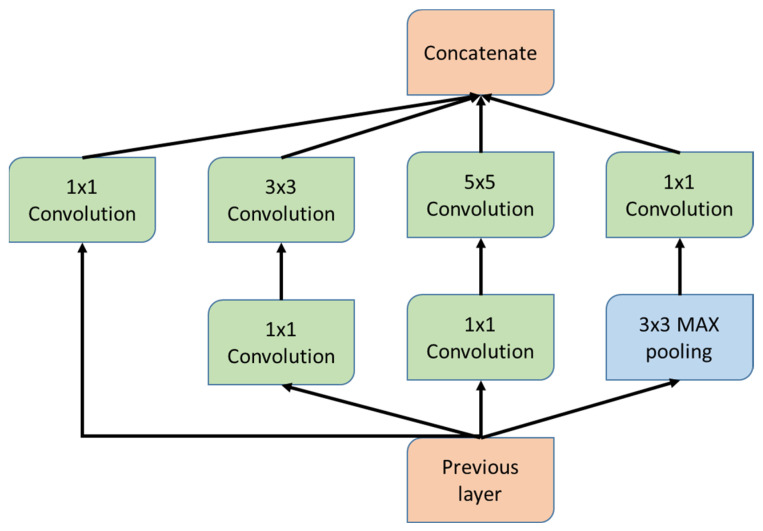
Inception module for the GoogleNet model.

GoogleNet [21] is a CNN that is 22 layers deep and efficiently uses the computational resources using repeated inception modules. These inception modules enhance the width and depth of the network that helps capture the features at varying scales. Each inception module contains different-sized convolutional layers to capture various local and spatial features of the input. The inception module for the GoogleNet model is shown in Figure 5. The 1 × 1 convolutional layers reduce the dimensions of the input and extract the local cross-channel features. The 3 × 3 and 5 × 5 convolutional layers help in capturing the spatial features of the input. The pooling layer is included in the inception module to reduce the dimensions of the input.

#### 3.2.2. Residual Networks (ResNet)

A Residual Neural Network (ResNet) [22] is a CNN that eliminates specific layers in the network using skip connections. The skip connections help solve the problem of vanishing gradients in the CNN and reduce the training time. Non-linear activation functions are used between the skipped layers. Batch normalization is also applied between the shortcut connections. A weight matrix is used that calculates the weights of the jump connections. After learning the features of the input, expansion is applied in the later stages of the network.

**Figure 6:**
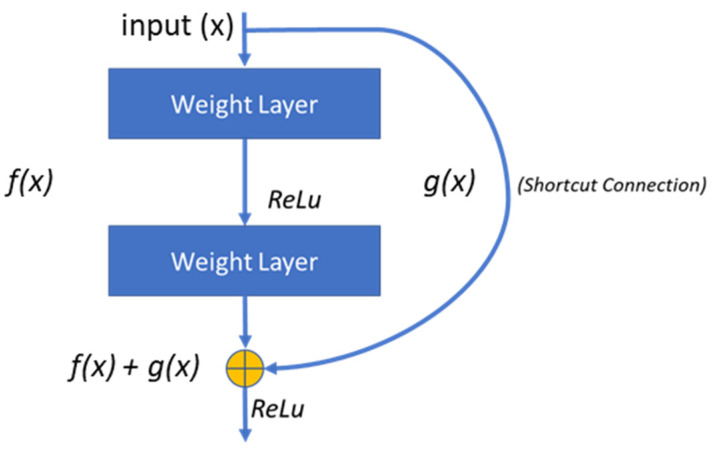
Building block of a residual network.

Figure 6 shows the basic building block of a ResNet. Multiple instances of the residual block are used throughout the network. In a CNN, the mapping from *x* → *f*(*x*) is learned. In the fundamental block of the residual network, the mapping is carried out by a feed-forward neural network that contains shortcut connections called jump or skip connections, i.e., *x* → *f*(*x*) *+ g*(*x*). The function *g*(*x*) is an identity connection if both the output and input dimensions match, otherwise, zero padding is applied.

The resulting residual block for the stacked layers in the network with the same dimensions can be given by Equation (3).

(3) $$y=f\left(x,\left\{W_{i}\right\}\right)+x$$

The function $f\left(x,\left\{W_{i}\right\}\right)$ represents the convolution layer mapping, which is learned during the training. The ResNet-18 CNN proposed in [22], uses 3 × 3 filters with a stride of 1, the average pooling layer contains 1 × 1 filter, and one fully connected layer is used at the end. This is followed by a final SoftMax layer for classification. The network contains a total of 17 convolutional layers with one fully connected layer at the end, which is reshaped to extract 1000 features in this work. Figure 1 shows the ResNet-18 model with a reshaped layer at the end for feature extraction.

#### 3.2.3. Transfer Learning

**Figure 7:**
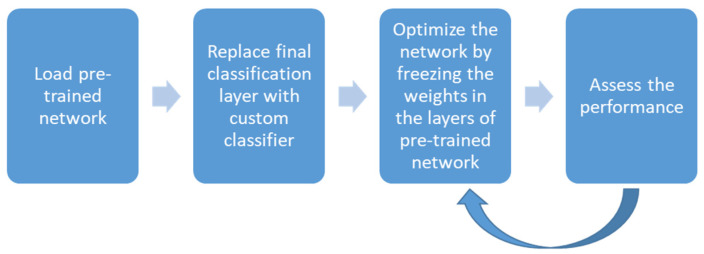
Workflow in transfer learning.

Transfer Learning is another field of deep learning in which learned features from one application’s model are transferred to a different application [38,39,40]. Transfer learning is useful when the input data are not substantial enough to train the CNN. In this method, pre-trained networks such as AlexNet [18], VGG [39], ResNet [22], GoogleNet [21], etc., are used to transfer the learned features of the model from a different system and apply the knowledge to a new set of input data. Different layers in the pre-trained CNN models are frozen and performance can be optimized. The general workflow in transfer learning is given in Figure 7.

### 3.3. Classification of Fundus Images

Machine learning algorithms classify images based on the features that are extracted from them. The main idea of image classification is the grouping of images with similar features. Linear or nonlinear combined image features are used in the classification process.

#### 3.3.1. Support Vector Machine (SVM)

An SVM is among the traditional classifiers and supervised machine learning algorithms [41]. The way that an SVM works is that it classifies the data input by forming a hyperplane in a higher dimension space. The process allows for applying a Kernel function to transform the input into hyperplanes, thus dividing the data into separate classes. SVM utilizes structural error minimization in the classification process and works to maximize the margins between the hyperplane classes. The different Kernel functions utilized by SVM include sigmoid function, hyperbolic tangent kernel, polynomial kernel, isotropic Gaussian kernel, etc.

#### 3.3.2. Random Forest (RF)

RF is another traditional classifier among the ensemble-based classifiers that work by combining different algorithms for the classification process [42]. Initially, randomly generated decision trees are formed together like a forest. The training set data are used to train all these trees. Another randomness with RF is that the data used for training are generated randomly. Bagging is a process within RF that prevents overfitting. Test features extracted after the initial creation of the forest are used in the final prediction of every output of the individual decision trees. The final vote of the decision tree is taken as the final output. After training, any new data are presented to the RF with the maximum vote used to determine the final output.

#### 3.3.3. Radial Basis Function (RBF)

Radial Basis Function (RBF) is another classifier that measures the similarity between the input data and training sample to determine the class [43]. A radial basis kernel is used to transform the n-dimensional input to a higher m-dimension. It is capable of generating a polynomial of infinite power allowing for the non-linear classification of the input data.

#### 3.3.4. Naïve Bayes (NB)

NB is yet another traditional classifier that is based on the probabilistic statistics model of the Bayes theorem [44]. The assumption that strong independence exists between the features of the images gives this classier the name of naïve. In the original Bayes classifier, the conditional probability of whether data belongs to a particular class is calculated through the conditional and unconditional probabilities of the same data belonging to each class within the dataset. The complexity of NB is finding the class within the data that has the same number of attributes with strong dependence.

## 4. Experimental Results

In binary classification, the data no longer suffer from the imbalance issue after combining stage 1–4 images into a single class, i.e., DR. For multiclass classification, we reduce the number of classes from five to three, i.e., NDR (stage 0), MDR (stage 1–2), and PDR (stage 3–4). We determine the smallest number of images in three classes and perform a randomized selection of the same number of images from the other classes. Using this method, we obtain the lowest number of images in the combination of stage 3–4 labeled class (PDR), i.e., 488. Hence, 488 images are randomly selected from each of the remaining classes, i.e., NDR and MDR. These images are passed on to the CNN models to extract the feature vectors. The batch size is set to 32. The feature vectors from the individual models are combined to form the hybrid feature vector, which is passed on to different classifiers. For additional comparison, the individual transfer learning models that use only the GoogleNet or ResNet-18 feature vector of 1000 features are also passed on to the classifiers.

The hardware used In this work contains an AMD Ryzen 2700× processor with 32 GB of RAM. The Graphics Processing Unit (GPU) installed in the system is an NVIDIA GeForce RTX 2080 with 8 GB memory. MATLAB was used for extracting the features of the pre-trained GoogleNet and ResNet-18 architectures based on APTOS image data. Different classifiers are used in MATLAB for binary and multiclass classification of input images.

The evaluation metrics which are used for assessing the performance of the system are accuracy, precision, recall, and f-measure. Accuracy represents the fraction of total predictions that are correctly classified. The precision determines what fraction of predictions classified as positive in a certain class are actually correct. Recall determines which fraction of actual correct labels in the data were predicted correctly by the classifier. F-measure provides the harmonic mean of recall and precision.

Table 1 shows the experimental results of applying binary classification on the feature vector extracted from the GoogleNet model. This feature vector is passed on to four classifiers, i.e., RF, SVM, RBF, and NB. Results show that the SVM classifier provides the highest individual class accuracy of 97.52% for the No DR class and 97.26% for the DR class. The average accuracy for SVM, i.e., 97.39%, is also the highest compared to other classifiers. SVM also achieves the highest average values for precision, recall, and f-measure at 97.40%.

In Table 2, the experimental results of applying binary classification on the feature vectors from the ResNet-18 model are presented. Similar to the GoogleNet model, results show that the SVM classifier provides the highest individual class accuracy of 97.25% for the No DR class and 98.08% for the DR class. The average accuracy for SVM, i.e., 97.67% is also the highest compared to other classifiers. SVM also achieves the highest average values for recall, f-measure, and precision at 97.70%. The lowest accuracy is achieved by the NB classifier with an average accuracy of 92.05%.

In Table 3, the results of using the proposed hybrid model, having a feature vector with 2000 features, are presented. This model achieves the highest average accuracy of 97.80% using the SVM classifier. The individual class accuracies are equal to the maximum class accuracies obtained between the ResNet-18 and GoogleNet models. Besides this metric, SVM is also able to achieve the highest average percentage in other metrics, i.e., precision, recall, and f-measure, at 97.8% each. The classifier with the lowest performance is the NB classifier with an average accuracy of 92.73%. NB also has the lowest average values of recall, f-measure, and at 92.7% each.

In Table 4, the results of multiclass classification using the features from the GoogleNet model are presented. The average accuracy of the SVM model is significantly lowered compared to binary classification because of interclass dependability between MDR and PDR classes. However, SVM still outperforms the other classifiers with an average accuracy of 79.95%. MDR class has the lowest individual accuracy of 68.98 in the SVM classifier. Other metrics of the SVM classifier still outperform others with a precision of 80.20%, recall of 80%, and f-measure of 80%.

The results of using the ResNet-18 model features for multiclass classification are presented in Table 5. SVM model still outperforms the other classifiers. The individual class accuracy of NDR is the same in SVM classifier as that of GoogleNet. The overall average accuracy of SVM is 77.44%, which is slightly lower than when using the GoogleNet model. However, the features extracted from the ResNet-18 model provide better classification accuracy for the PDR class.

Making a hybrid set of features extracted from both models provides significant improvements in the classification, as depicted in Table 6. The SVM classifier outperforms others and provides the highest average class accuracy of 89.29% using the hybrid features vector. The individual class accuracies are also the highest reported for multiclass SVM classifiers with NDR at 96.66%, MDR at 81.64%, and PDR at 90.07%. The performance of the remaining parameters is also reported at the highest values with an average precision of 89.40%, average recall of 89.30%, and average f-measure of 89.30%.

**Figure 8:**
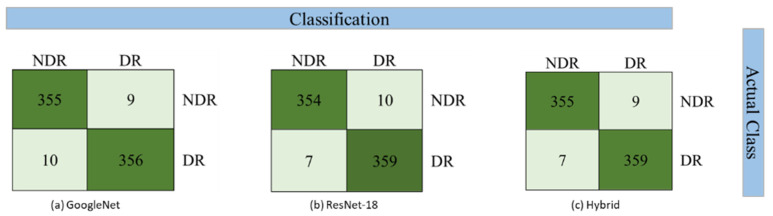
Confusion matrix for binary classification using SVM classifier.

**Figure 9:**
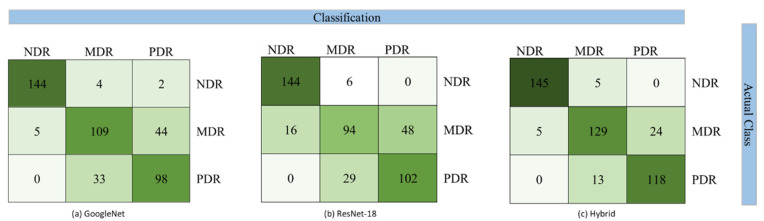
Confusion matrix for multiclass classification using SVM classifier.

The confusion matrix for binary classification using the SVM classifier is depicted in Figure 8. For multiclass classification, the confusion matrix is presented in Figure 9.

## 5. Discussion

In this work, we presented a hybrid feature extraction method using transfer learning and used different classifiers for detecting DR in fundus images. The proposed model is able to effectively classify fundus images into different stages of DR. The results, when compared with recent research articles, provide significant improvement in the average accuracy of DR detection. The comparison of work with recent research articles is presented in Table 7. The maximum average accuracy achieved in this paper using hybrid features vector and SVM classifier is 97.80% for binary classification and 89.29% for multiclass classification. The closest average accuracy for binary classification is reported in [29] at 97.00%, whereas for multiclass classification, the closest is reported in the article [23] with a value of 82.18%. When performing classification using the features extracted from GoogleNet only (1000 features), the MDR class provides better performance compared to the ResNet-18 model. Similarly, the features from the ResNet-18 model outperform the GoogleNet model for PDR class metrics. Merging the two feature vectors gives an optimal performance in classifying both the PDR and MDR classes which increases the overall efficiency of the system. The proposed system achieves the classification in minimal time since the feature vectors are extracted from pre-trained models of GoogleNet and ResNet-18. Another possible approach that needs to be studied in the future is the effect of designing a customized CNN that can fully capture the interclass similarities between all the classes of DR while maintaining high-performance metrics. Besides DR, other diseases such as age-related macular degeneration and glaucoma can cause irreversible damage to the retina. Automated detection of these using different machine learning and deep learning techniques on fundus images and optical coherence tomography-based images needs to be worked on in the near future [45,46,47].

## 6. Conclusions

This research work provides a hybrid approach for early Diabetic Retinopathy detection using transfer learning to extract fundus image features from ResNet-18 and GoogleNet models. These features are input to different classifiers which perform binary and multiclass classification of DR images from the APTOS dataset. In multiclass classification, the combination of features extracted from GoogleNet and ResNet-18 help improve the MDR and PDR class metrics that increase the overall performance of the system. The proposed classification technique can assist ophthalmologists in the early detection of Diabetic Retinopathy. The results also indicate that using CNN for feature extraction followed by other machine learning classifiers besides ANN can provide fast and highly accurate results. The hybrid model using the SVM classifier achieves the highest average accuracy of 97.80% for binary classification and 89.29% for multiclass classification. The results outperform recent similar approaches to binary and multiclass DR detection.

Future work will continue in the line of detection and diagnosis of Diabetic Retinopathy using various machine learning algorithms and using deep learning algorithms. Enhancements can be carried out to improve the results such as data augmentation and applying different preprocessing techniques to remove different artifacts and noise from the input images. This very important area of research will remain open for continuous improvement.

## Figures and Tables

**Table 1:** Experimental results of different classifiers for binary classification using features extracted from the GoogleNet Model.

Classifier	Metrics	NDR	DR	Weighted Average
RF	Accuracy	95.32	95.90	95.61
	Precision	95.90	95.40	95.60
	Recall	95.30	95.90	95.60
	F-Measure	95.60	95.60	95.60
SVM	Accuracy	97.52	97.26	97.39
	Precision	97.30	97.50	97.40
	Recall	97.50	97.30	97.40
	F-Measure	97.40	97.40	97.40
RBF	Accuracy	96.70	97.26	96.98
	Precision	97.20	96.70	97.00
	Recall	96.70	97.30	97.00
	F-Measure	97.00	97.00	97.00
NB	Accuracy	89.83	85.79	87.80
	Precision	86.30	89.50	87.90
	Recall	89.80	85.80	87.80
	F-Measure	88.00	87.60	87.80

**Table 2:** Experimental results of different classifiers for binary classification using features extracted from the ResNet-18 Model.

Classifier	Metrics	NDR	DR	Weighted Average
RF	Accuracy	95.60	94.26	94.93
	Precision	94.30	95.60	94.90
	Recall	95.60	94.30	94.90
	F-Measure	95.00	94.90	94.90
SVM	Accuracy	97.25	98.08	97.67
	Precision	98.10	97.30	97.70
	Recall	97.30	98.10	97.70
	F-Measure	97.70	97.70	97.70
RBF	Accuracy	97.25	96.17	96.71
	Precision	96.20	97.20	96.70
	Recall	97.30	96.20	96.70
	F-Measure	96.70	96.70	96.70
NB	Accuracy	89.83	94.26	92.05
	Precision	94.00	90.30	92.10
	Recall	89.80	94.30	92.10
	F-Measure	91.90	92.20	92.10

**Table 3:** Experimental results of different classifiers for binary classification using hybrid features extracted GoogleNet and ResNet-18.

Classifier	Metrics	NDR	DR	Weighted Average
RF	Accuracy	96.42	95.62	96.02
	Precision	95.60	96.40	96.00
	Recall	96.40	95.60	96.00
	F-Measure	96.00	96.00	96.00
SVM	Accuracy	97.52	98.08	97.80
	Precision	98.10	97.60	97.80
	Recall	97.50	98.10	97.80
	F-Measure	97.80	97.80	97.80
RBF	Accuracy	97.25	97.26	97.26
	Precision	97.30	97.30	97.30
	Recall	97.30	97.30	97.30
	F-Measure	97.30	97.30	97.30
NB	Accuracy	92.30	93.16	92.73
	Precision	93.10	92.40	92.70
	Recall	92.30	93.20	92.70
	F-Measure	92.70	92.80	92.70

**Table 4:** Experimental results of different classifiers for multiclass classification using features extracted from the GoogleNet Model.

Classifier	Metrics	NDR	MDR	PDR	Weighted Average
RF	Accuracy	94.66	68.35	70.99	78.13
	Precision	91.60	73.00	68.40	78.00
	Recall	94.70	68.40	71.00	78.10
	F-Measure	93.10	70.60	69.70	78.00
SVM	Accuracy	96.00	68.98	74.80	79.95
	Precision	96.60	74.70	68.10	80.20
	Recall	96.00	69.00	74.80	80.00
	F-Measure	96.30	71.70	71.30	80.00
RBF	Accuracy	96.00	58.86	75.57	76.53
	Precision	91.70	73.80	63.50	76.80
	Recall	96.00	58.90	75.60	76.50
	F-Measure	93.80	65.50	69.00	76.20
NB	Accuracy	87.33	62.02	67.17	72.20
	Precision	84.00	65.30	66.20	72.00
	Recall	87.30	62.00	67.20	72.20
	F-Measure	85.60	63.60	66.70	72.10

**Table 5:** Experimental results of different classifiers for multiclass classification using features extracted from the ResNet-18 Model.

Classifier	Metrics	NDR	MDR	PDR	Weighted Average
RF	Accuracy	96.00	58.22	75.57	76.30
	Precision	87.30	72.40	67.30	76.00
	Recall	96.00	58.20	75.60	76.30
	F-Measure	91.40	64.60	71.20	75.70
SVM	Accuracy	96.00	59.49	77.86	77.44
	Precision	90.00	72.90	68.00	77.30
	Recall	96.00	59.50	77.90	77.40
	F-Measure	92.90	65.50	72.60	77.00
RBF	Accuracy	98.66	52.53	80.15	76.53
	Precision	88.10	76.10	64.80	76.80
	Recall	98.70	52.50	80.20	76.50
	F-Measure	93.10	62.20	71.70	75.60
NB	Accuracy	90.00	61.39	71.75	74.25
	Precision	86.50	68.80	66.20	74.10
	Recall	90.00	61.40	71.80	74.30
	F-Measure	88.20	64.90	68.90	74.10

**Table 6:** Experimental results of different classifiers for multiclass classification using hybrid features extracted GoogleNet and ResNet-18.

Classifier	Metrics	NDR	MDR	PDR	Weighted Average
RF	Accuracy	96.66	76.58	83.96	85.64
	Precision	92.40	84.00	79.70	85.60
	Recall	96.70	76.60	84.00	85.60
	F-Measure	94.50	80.10	81.80	85.50
SVM	Accuracy	96.66	81.64	90.07	89.29
	Precision	96.70	87.80	83.10	89.40
	Recall	96.70	81.60	90.10	89.30
	F-Measure	96.70	84.60	84.60	89.30
RBF	Accuracy	98.66	62.65	82.44	80.86
	Precision	93.70	81.10	67.90	81.50
	Recall	98.70	62.70	82.40	80.90
	F-Measure	96.10	70.70	74.50	80.50
NB	Accuracy	94.00	74.68	71.75	80.41
	Precision	89.80	74.70	75.80	80.20
	Recall	94.00	74.70	71.80	80.40
	F-Measure	91.90	74.70	73.70	80.30

**Table 7:** Comparison of proposed hybrid model with recent research articles.

Reference	Method	Dataset	Accuracy
Proposed Method	Transfer Learning-based Hybrid GoogleNet and ResNet-18 features with SVM Classifier	APTOS	97.80% (Binary), 89.29% (Multiclass)
Farag et al. (2022) [29]	DenseNet with Convolutional Block Attention Module	APTOS	97.00% (Binary)
Vives Bois (2021) [30]	convolutional neural networks with synaptic metaplasticity	APTOS	94.00% (Binary)
Zhang (2022) [31]	Source-Free Transfer Learning Approach	APTOS	91.2% (Binary)
Gangwar et al. (2021) [23]	Transfer Learning with additional CNN layers in the ResNet model	APTOS	82.18% (Multiclass)

## Data Availability

Not applicable.
